# Suppression of acute pancreatitis by L-lysine in mice

**DOI:** 10.1186/s12906-015-0729-x

**Published:** 2015-06-23

**Authors:** Abdulrahman L. Al-Malki

**Affiliations:** Department of Biochemistry, Faculty of Science, King Abdulaziz University, P.O. Box 80203, Jeddah, 21589 Saudi Arabia

**Keywords:** Pancreatitis, Lysine, Rats

## Abstract

**Background:**

Acute pancreatitis is an inflammatory disease caused by several factors such as viral infection, drugs, and diagnostic endoscopy. The aim of this study was to evaluate the potential protective or therapeutic effects of L-lysine on pancreatitis induced by L-arginine in mice.

**Methods:**

Four groups of mice (10 in each group) were assessed. Group I was the control. Animals in groups II–IV were injected intraperitoneally with L-arginine hydrochloride (400 mg/kg body weight [bw]) for 3 days. Group III animals were orally pre-treated with L-lysine (10 mg/kg bw), whereas group IV animals were orally post-treated with L-lysine (10 mg/kg bw). Serum samples were subjected to amylase, lipase, transaminase, and interleukin-6 (IL-6) assays. The pancreas was excised to measure the levels of malondialdehyde, nitric oxide, catalase, superoxide dismutase, reduced glutathione, and glutathione peroxidase.

**Results:**

Pre- or post-treatment with L-lysine led to significant decreases in the levels of malondialdehyde and nitric oxide, while significant enhancement was observed in the activities of antioxidant enzymes (superoxide dismutase, catalase, and glutathione peroxidase) and glutathione (*p* < 0.001). However, the treatment potential of L-lysine was better as a protective agent than a therapeutic agent.

**Conclusions:**

L-lysine treatment attenuates pancreatic tissue injury induced by L-arginine by inhibiting the release of the inflammatory cytokine IL-6 and enhance antioxidant activity. These effects may involve upregulation of anti-inflammatory factors and subsequent downregulation of IL6.

## Background

Acute pancreatitis is identified by local necrotic pancreatic tissue and systemic functional failure, and is highly correlated with an increase in mortality [1]. Acute pancreatitis may be caused by several factors including alcoholism, viral, bacterial or parasitic infection as well as diagnostic procedures such as endoscopy [2]. During acute pancreatitis, intracellular enzymes such as amylase and lipase are released, leading to elevations in their serum levels. Serum amylase peaks within hour post-pancreatitis [3] and remain steady for 2–4 days before returning to normal. However, pancreatic lipase is more sensitive to degradation and remains elevated for 10 days [4].

A recent study has reported additional markers for acute pancreatitis, including C-reactive protein, α1-antitrypsin, phospholipase A_2,_ and inflammatory markers such as interleukin-6 (IL-6) [5].

Experimental induction of pancreatitis has been developed to evaluate recovery and treatment strategies [6]. It has been reported that injection of L-arginine (300 mg/100 g body weight [bw], intraperitoneal [i.p.]) into rats damages pancreatic cells. Based on this observation, such an approach has been used to experimentally model pancreatitis [7].

There are different studies which develop acute experimental pancreatitis including arginine induced pancreatitis, cerulein via secretagogue induced pancreatitis, duct obstruction induced pancreatitis and vascular induced pancreatitis [8].

A previous study has reported that i.p. administration of excessive doses of L-arginine (500 mg/100 g bw) to rats leads to selective damage of pancreatic acinar cells without any morphological change in the islets of Langerhans or other organs [9]. Subsequently, Tani et al [9] reported an L-arginine-induced rat model of necrotizing acute pancreatitis. Doses of L-arginine higher than 500 mg/100 g bw caused very high mortality. A single L-arginine dose of 500 mg/100 g bw injected *i.p.* resulted in 70–80 % pancreatic acinar cell necrosis within 3 days. Since these observations, the rat model of L-arginine-induced acute pancreatitis has been applied in various studies with variations in the dose and frequency of administration. The advantages of uses arginine in induction of pancreatitiss due to its high reproducibility, ability to achieve selective dose-dependent pancreatic acinar cell necrosis, suitable for investigating the early and late phases of acute pancreatitis and useful for investigating the insulo-acinar axis [10, 11].

Most commonly L -arginine in doses of 200–600 mg/kg are used to induce experimental acute pancreatitis in rats. Thus, the basic amino acid-induced models seem to morphologically resemble human necrotizing pancreatitis in which nerves, major ducts and islets are not markedly affected [12]. One important drawback of the basic amino acid-induced models is that extra cute pancreatitits ancreatic complications due to acute pancreatitis are mild in these models. In mice, the effective and toxic/lethal doses of basic amino acids are very close to each other and toxicity, which is frequently lethal, is most likely to be due to metabolic effects of the basic amino acids themselves rather than to the associated acute pancreatitis. Rats and mice become lethargic soon after the i.p. injection of amino acids and it takes several hours for the animals to recover from this phase. The mortality associated with the L-arginine model in mice has been reported [12].

The catabolic state induced by the response to injury in acute pancreatitis lead to increased breakdown of endogenous protein and 40 % of free amino acids significantly decreased. For this reason the rational of this study improve nitrogen balance by supplementation of essential amino acid. L-lysine is an essential amino acid with important roles in connective tissues and carnitine synthesis, energy production, growth in children, and maintenance of immune functions [13, 14]. This study was designed to evaluate the potential protective or therapeutic effects of L-lysine on L-arginine-induced acute pancreatitis in mice.

## Methods

### Animals

Forty male mice weighing 40–60 g were used in this study. They were housed under standard conditions and allowed access to water and a standard diet *ad libitum*. The animal protocol was approved by the King AbdulAziz University Animal Ethics committee. The animals were divided into four groups with 10 animals in each group. Group I animals served as controls. Animals in groups II–IV were *i.p.* injected with L-arginine hydrochloride (400 mg/100 g bw, dissolved in saline, pH 7) for 3 days to induce pancreatitis as described by Tani etal [9]. Group III animals were orally pre-treated with L-lysine (10 mg/kg bw) (Sigma-Aldrich, StLouis, MO), whereas group IV animals were post-treated orally with L-lysine (10 mg/kg bw). After 15 days, the animals were anesthetized with 10 % thiopental. Blood was collected without anticoagulation, and serum was separated by centrifugation at 4000*x* g for 15 min. The pancreas was excised immediately and homogenized in ice-cold 0.1 M Tris-HCl. The pancreatic homogenate was used to measure oxidative stress markers.

### Amylase, lipase, alanine transaminase (ALT), and aspartate transaminase (AST) assays

Serum amylase, lipase ALT, and AST were assayed using commercially available kits from Bio Vision Research Products (Avenue, USA) according to the manufacturer’s instructions.

### IL-6 assay

Serum IL-6levelswere assayed using an ELISA kit from R&D (Berlin, Germany) according to the manufacturer’s instructions. Protein concentrations of liver homogenate swere assayed using a BCA mega kit (Pierce, Rockford, USA).

### Malondialdehyde (MDA)assay

MDA was assayed using a method described elsewhere [15]. The concentration of MDA was calculated and expressed as n mol.mg^−1^ protein.

### Measurement of nitric oxide (NO) concentrations

NO formation was determined by an indirect method by quantifying the tissue level of nitrite using a calorimetric method based on the Griess reaction [16] using a kit from Bio-Rad (UK).

### Super oxide dismutase (SOD) activity assay

The activity of SOD was assayed as described elsewhere [17] using a kit from Bio-Rad. SOD activity was expressed as U/mg protein.

### Determination of glutathione peroxidase activity

The method of Paglia and Valentine [18] was used to measure the activity of glutathione peroxidase in liver homogenates using a kit from Bio-Rad. Glutathione peroxidase activity was expressed as U/mg protein.

### Reduced glutathione (GSH) assay

GSH was assayed by the colorimetric end point method as described elsewhere [19] using a kit from Bio-Rad.

### Determination of catalase activity

The method of Kakkar [20] was used to determine the activity of catalase in pancreatic homogenates using a kit from Bio-Rad. Catalase activity was expressed as U/mg protein.

### Histopathological examination

Pancreas tissue was collected from animals, washed carefully by cold normal saline 3 times, then fixed in formalin solution 10 %, processed, and embedded in a paraffin film. Sections of 5-μm thick were prepared. The sections were stained with H&E and examine by light bifocal microscope.

### Statistical analysis

Statistical analysis was performed using one-way analysis of variance (ANOVA) followed spearman correlation test. Data are presented as the arithmetic mean ± standard deviation (SD). The differences among means were analyzed by one-way analysis of variance. A value of *P* < 0.05 was considered as statistically significant.

## Results

Animals injected with arginine (400 mg/kg bw) showed a significant elevation in serum amylase, lipase, and transaminases (ALT and AST) activities compared with the control group (*P* < 0.001; Table 1). However, L-lysine treatment led to a significant reversal in the elevation of these enzyme activities (*P* < 0.05), but not returned to normal levels. The results indicated that pre-treatment with L-lysine was more effective than post-treatment. Serum IL-6 levels were significantly increased in arginine-injected mice (*P* < 0.001) compared with the control group, whereas L-lysine treatment modulated this elevation (*P* < 0.01). However, L-lysine pre-treatment was more effective than post-treatment. A positive correlation was found between transaminases and IL-6 (r = 0.67).

**Table 1:** Activities of amylase, lipase, alanine transaminase (ALT), aspartate transaminase (AST), and interleukin-6 (IL-6) levels in serum samples

Group:	I	II	III	IV
Parameter	(Normal)	(Arginine, 400 mg/kg)	(Arginine + Lysine) treated	(Arginine + Lysine) protected
Amylase (U/l)				
Mean ± SD	400 ± 45	1600 ± 99	1191 ± 84	650 ± 75
*P* value	---	<0.001	<0.001	<0.001
P*		---	<0.001	<0.001
Lipase (U/l)				
Mean ± SD	127 ± 19	397 ± 76	211 ± 57	180 ± 79
*P* value	---	<0.001	<0.001	<0.001
P*		--	<0.01	<0.01
AST(U/l)				
Mean ± SD	25 ± 2.3	64 ± 2.0	46 ± 2.5	33 ± 9
P value		<0.001	<0.001	<0.001
P*			<0.05	<0.05
ALT(U/l)				
Mean ± SD	24 ± 3.1	50.7 ± 4.2	42 ± 2.2	36 ± 2.6
*P* value	---	<0.001	<0.001	<0.001
P*		--	<0.05	<0.001
IL-6 (ng/l)				
Mean ± SD	22 ± 2.1	90.7 ± 7.7	63 ± 5.1	39 ± 5
*P* value	---	<0.05	<0.05	<0.001
P*		--	<0.05	<0.01

*P* < 0.05 compared with the control

* Treated versus untreated

Measurements of oxidative stress markers in pancreas samples (Table 2) showed elevations in the levels of MDA and NO (*P* < 0.05), indicating that the mice underwent increases in free radical production and oxidative damage. Pre -or post-treatment. With L-lysine significantly decreased the levels of MDA and NO while significantly increasing total antioxidant activity (*P* < 0.05) (Table 2).

**Table 2:** Oxidative stress markers, malondialdehyde (MDA) and nitric oxide (NO), and antioxidant enzyme activities in pancreatic tissues

Group:	I	II	III	IV
Parameter	(Normal)	(Arginine, 400 mg/kg)	(Arginine + Lysine) Treated	(Arginine + Lysine) Protected
MDA (mmol/mg protein)				
Mean ± SD	48 ± 4.5	107 ± 13	81 ± 10	59 ± 5.1
*P* value	---	<0.001	<0.01	<0.001
P*		---	<0.001	<0.001
NO (ug/mg protein)				
Mean ± SD	5.9 ± 0.9	19 ± 2.3	11.0 ± 2	9 ± 0.6
*P* value	---	<0.001	<0.001	<0.001
P*		--	<0.001	<0.001
SOD (U/ mg protein)				
Mean ± SD	1225 ±	798 ± 96	869 ± 102	998 ± 152
*P* value	203	<0.001	<0.001	<0.001
P*			<0.001	<0.001
GPx (U/ mg protein)				
Mean ± SD	733 ± 56	1450 ± 101	1100 ± 92	980 ± 122
*P* value	---	<0.05	<0.05	<0.001
P*		--	<0.05	<0.001
Reduced glutathione (ug/ mg				
protein)	222 ± 21	97 ± 11	133 ± 32	175 ± 35
Mean ± SD	---	<0.05	<0.05	<0.01
*P* value		--	<0.05	<0.01
P*				
Catalase (U/ mg protein)				
Mean ± SD	96 ± 10.3	65 ± 7	63 ± 2.2	73 ± 2.5
*P* value	---	<0.05	<0.01	<0.01
P*		--	<0.01	<0.01

SOD, super oxide dismutase; GPx, glutathione peroxidase; *P* < 0.05 compared with the control

* Treated versus untreated

In mice injected with arginine, we found that the activities of antioxidant enzymes (SOD and catalase) and GSH levels were decreased significantly (*p* < 0.001), and a significant increase in the activity of glutathione peroxidase (*p* < 0.001). Pre-or post-treatment with L-lysine reversed these changes to near normal levels. A negative correlation between the level of MDA and SOD and catalase (r = 0.54 and 0.76) respectively.

### Histological examination

**Fig. 1:**
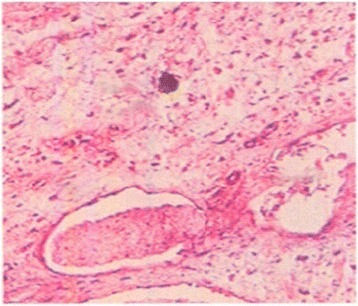
Control pancreas

**Fig. 2:**
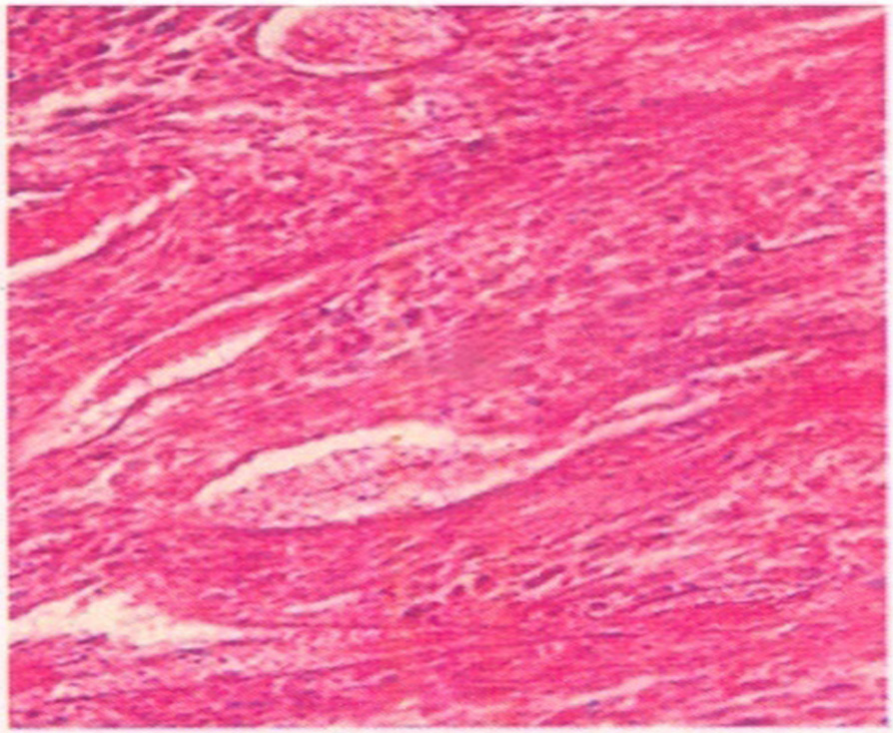
Pancreatitis

**Fig. 3:**
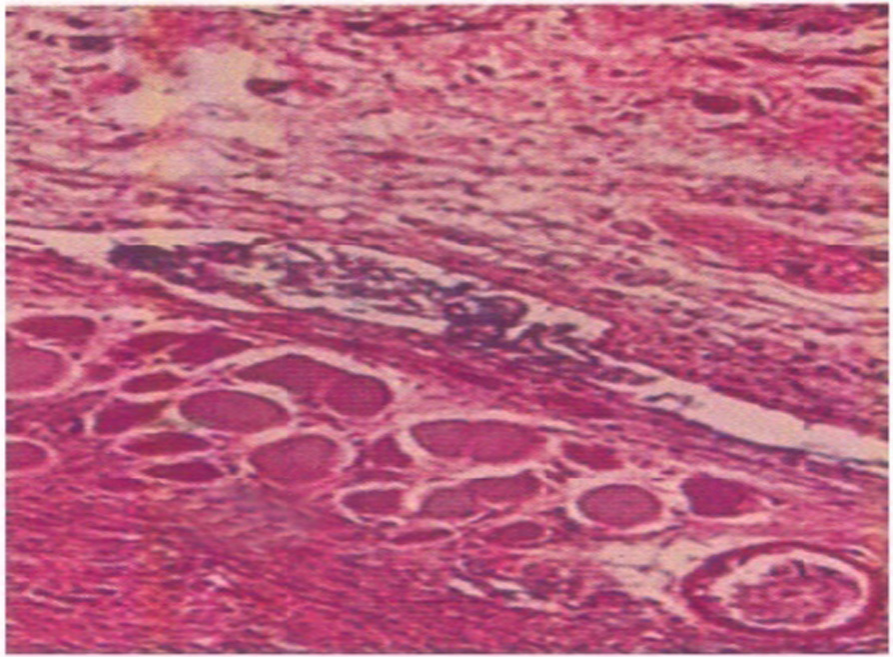
Pancreatitis post-treated with lysine

**Fig. 4:**
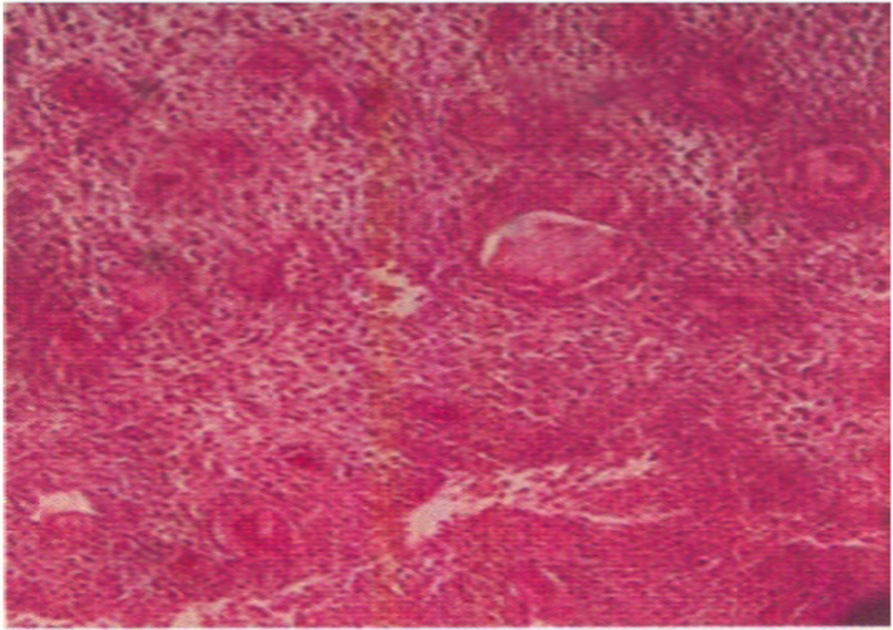
Pancreatitis pre-treated with lysine

Compared with the control group (score: 1.0 ± 0.1, Fig. 1), the pancreas in the arginine-injected group (6.2 ± 1, Fig. 2) showed hemorrhaging and the presence of neutrophils and macrophages. The acini were expanded and necrotic. Mice post-treated (Fig. 3) or pre-treated (Fig. 4) with L-lysine showed decreases in the necrosis rate and inflammatory cell infiltration (*P* < 0.01) (scores: 4.0 ± 0.8 and 2.9 ± 0.4, respectively).

## Discussion

Nutritional depletion of essential nutrient s have implicated in the etiology of many organ dysfunction. In the experimental rodents, L-arginine induced partial inhibition of L-arginase enzyme, which converts L-arginine to L-ornithine and urea [21]. High dose of L-ornithine has also been implicated to cause acute pancreatitis [22]. L-arginine is precursor of nitric oxide and nitrate which are known to induce oxidative stress. It was reported that L-arginine could induce pancreatitis through activation of neurotransmitters synthesis. It was found that, *i.p.* administration of L-arginine resulted in metabolic acidosis and reduction of pH of urine samples reaching the most acidic values (pH = 5.5–6.0). The production of oxygen free radicals occurs early in acute pancreatitis, overwhelming the antioxidant defense system. The acinar cells significantly contribute to the generation of large amounts of oxygen free radicals at the early stages of acute pancreatitis.

Our results demonstrated that i.p. injection of 400 mg/kg bw arginine induced acute pancreatitis and neutrophil infiltration in the pancreas as indicated by elevations in serum amylase, lipase, and transaminase activities, in addition the IL-6 level compared with the control. Pre- or post-treatment with L-lysine reversed these changes (*P* < 0.01). Lysine is antagonistic to arginine and competes with arginine for absorption, re-absorption, and transport across the cell membrane. *In vitro*, lysine inhibits the growth-promoting action of arginine as a potential benefit in managing arginine pancreatitis [23].

L-lysine protected the pancreas from injury by inhibiting the release of the inflammatory cytokine IL-6. The mechanism by which L-lysine exerts an anti-inflammatory effect may be inhibition IL-6 expression, a key regulator of inflammatory mediator expression, as an early event in experimentally induced pancreatitis, which correlates with the inflammatory response. In addition, L-lysine inhibited lipid peroxidation by decreasing MDA production through the release of IL-6. This observation is in agreement with our results. MDA is a marker of lipid peroxidation, and NO levels were significantly elevated while antioxidants were significantly decreased in arginine-injected mice compared with the control group. While the activities of antioxidant enzymes, including SOD, catalase, glutathione peroxidase, were significantly increased in response to L-lysine, indicating that L-lysine is an indirect antioxidant. These results were supported by the histological examination revealing an improvement in the arrangement of cells, normal nuclei, and decreases in macrophage and neutrophil infiltration.

Recent study reported that cytokines as IL-6 released from the inflamed pancreas can induce the synthesis of the inducible nitric oxide synthase, resulting in elevation of free NO, which serve as a key cellular mediator of inflammation [22]. This finding is in accordance with our results showing a reversal of pancreatitis by L-lysine administration.

In this study, the level of NO was significantly higher in the arginine-treated group compared with that in the control group (*P* < 0.001). NO is a highly reactive free radical that is produced from L-arginine by a family of NO syntheses. Moreover, the released NO react with superoxide causes the formation of peroxynitrite, which is a potent oxidant agent that play an important role in the cellular damage. These findings are reflected by the decrease of SOD activity in arginine-treated group and reversal by L-lysine treatment. The binding of peroxynitrite with proteins led to nitration of tyrosine to form nitrotyrosine, which is a specific a marker for peroxynitrite induced oxidative tissue damage. It is thus evident that the increase of NO concentration is related to the lesion of pancreas and other organs. In addition, the activity of SOD as an antioxidant was significantly decreased and that of MDA as a lipid peroxidase was significantly increased, which further indicated that free radical production and oxidation were intensified by arginine and reversed by lysine administration.

Since cytokines are found to induce oxidative stress by the generation of oxygen free radicals, iNOS expression, NO and superoxide production. Cytokine resulted in a large increase in iNOS protein levels. This increment was inhibited by administration of L-Lysine. As a consequence the amounts of NO was significant decreased. L-Lysine availability was inversely proportional to superoxide production perhaps as a result in suppression of pancreatitis.

The positive actions of L-lysine on antioxidant status is likely to reflect, in large part, enhancement of GSH and/peroxidase systems as the main line of antioxidant defense. GSH,may regenerate it from GSSG via a GSSG reductase catalyzed reaction. The net action of L-Lysine is to remove free radicals and protect against oxidative damage of these radicals.

Lysine treatment significantly decreased the histopathological scores and protected against elevation of serum amylase, ALT, AST activities, and IL-6 level. Pancreatic injury and hydrolytic enzyme release activate pro-inflammatory cells, resulting in the production of inflammatory mediators as cytokines, which cause a systemic multiple organ injury [24, 25].

## Conclusion

It was concluded that, lysine attenuates pancreatic tissue injury induced by L-arginine by inhibiting the release of the inflammatory cytokine as IL-6 and enhance antioxidant capacity. These effects may involve upregulation of anti-inflammatory mediators and antioxidants capacity and subsequent downregulation of IL-6.
